# Synthesis and characterisation of microcapsules for self-healing dental resin composites

**DOI:** 10.1186/s12903-023-03764-8

**Published:** 2024-01-18

**Authors:** Khaled Abid Althaqafi, Julian Satterthwaite, Abdulrahman AlShabib, Nikolaos Silikas

**Affiliations:** 1 Faculty of Dentistry, College of Dental Medicine, University of Umm Al Qura, Makkah, Kingdom of Saudi Arabia; 2 Division of Dentistry, School of Medical Sciences, University of Manchester, Oxford Road, Manchester, M13 9PL UK; 3 Department of Restorative Dentistry, College of Dentistry, King Saud University, Riyadh, Saudi Arabia

**Keywords:** Self-healing, Microcapsules, Resin composite, Dental composite

## Abstract

**Aim:**

The purpose of this study was to i) synthesise TEGDMA-DHEPT microcapsules in a laboratory setting; ii) characterise the resultant microcapsules for quality measures.

**Materials & methods:**

Microcapsules were prepared by in situ polymerization of PUF shells. Microcapsules characterisation include size analysis, optical and SEM microscopy to measure the diameter and analyse the morphology of PUF microcapsules. FT-IR spectrometer evaluated microcapsules and benzyl peroxide catalyst polymerization independently.

**Result:**

Average diameter of TEGDMA-DHEPT microcapsules was 120 ± 45 μm (*n*: 100). SEM imaging of the capsular shell revealed a smooth outer surface with deposits of PUF nanoparticles that facilitate resin matrix retention to the microcapsules upon composite fracture. FT-IR spectra showed that microcapsules crushed with BPO catalyst had degree of conversion reached to 60.3%.

**Conclusion:**

TEGDMA-DHEPT microcapsules were synthesised according to the selected parameters. The synthesised microcapsules have a self-healing potential when embedded into dental resin composite as will be demonstrated in our future work.

**Graphical Abstract:**

Graphical abstract showing the microcapsule components. The shell contains poly(urea-formaldehyde), and the core consists of TEGDMA-DHEPT healing agents. 
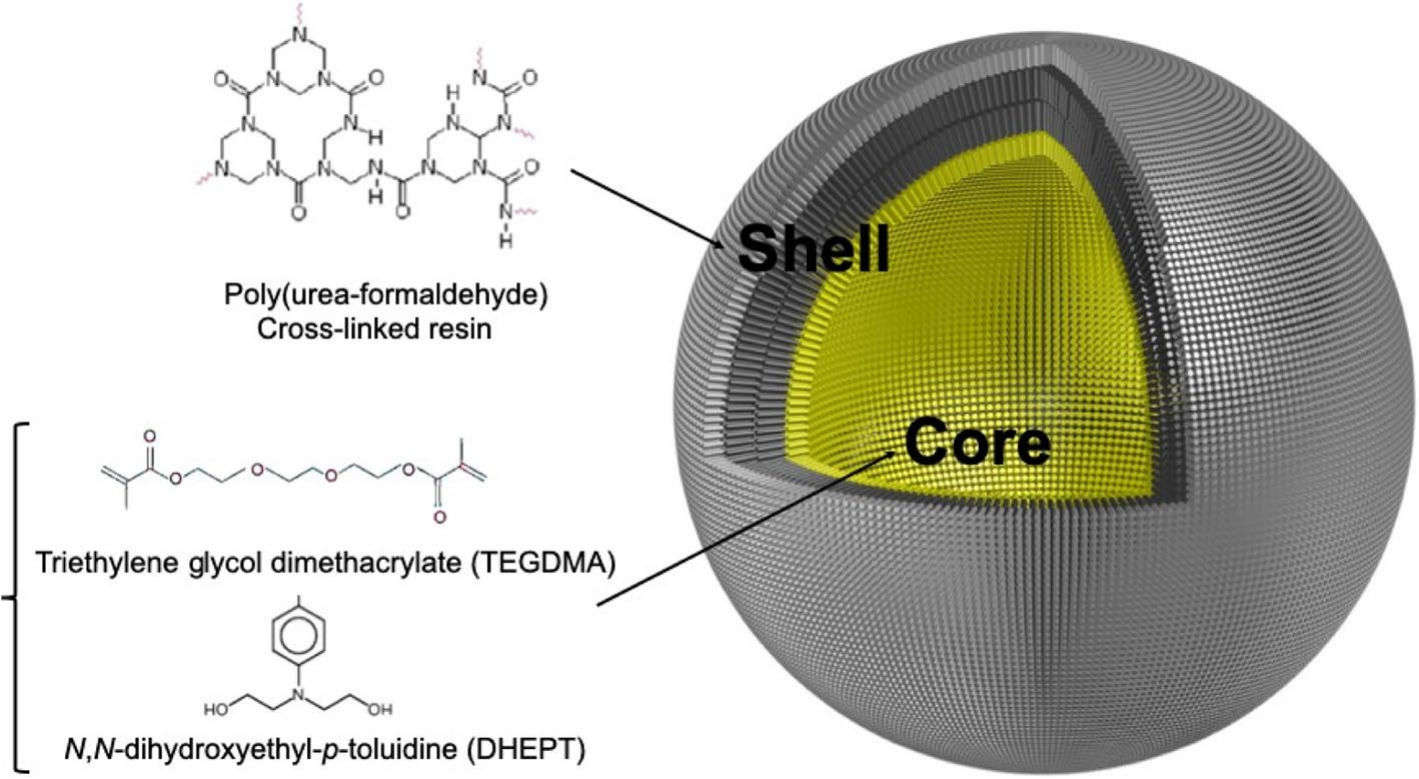

## Introduction

Dental resin restorations have been shown to encounter two main downsides: secondary caries and bulk fractures [1]. They frequently fail due to the accumulation of micro-cracks generated from masticatory forces and thermal stresses [2]. Self-healing composites and polymers involving microencapsulated healing liquids demonstrate the capability for providing long-life structural materials, with the potential to repair crack damage and recover mechanical performance in polymeric materials [3].

Self-healing systems to date are bioinspired from autonomic repair mechanisms in living tissues such as broken bone healing or/and soft tissue healing processes, with an effort to mimic natural components and implement self-healing capabilities into resin polymers [4–8]. Self-healing mechanisms have been achieved in bulk thermosetting polymers [9–13], self-healing fibre-reinforced composites [14–19], self-healing dental resin composites [20–24], self-healing adhesives [25], self-healing bonding resins [26], elastomers [27, 28] and coatings [29]. Self-healing approaches may involve hollow fibres [16, 17, 30, 31], nano-fibres [32, 33], microcapsules [3, 12, 14, 20, 22, 23, 34–36], nano-capsules [26, 37–39], or solid-state repairable polymers (heat application) [40, 41].

Microencapsulation is defined as “a technology of packaging solids, liquids or gaseous materials in miniature, sealed capsules that can release their contents at controlled rates under the influence of specific conditions” [42]. This is a promising approach to increasing the durability of resin composites as the microcapsules will rupture and release of polymerisable healing agents to seal and stop crack propagation when the composite is subjected to fracture [3, 14, 22].

Technological advances in microencapsulation methods have been introduced; allowing a higher level of standardisation in microencapsulation process. Published reports of lab-based microcapsules synthesis vary but include microfluidic (micro-channels) encapsulation method, mostly used in biological substances, controlled drug release and pharmaceuticals [43–45]. Although a higher precision can be achieved with microfluidics system, the microchannels are at a higher risk of clogging by PUF shell polymerization during microencapsulation which might be a limiting factor.

Studies have reported the incorporation of poly(urea-formaldehyde) PUF microcapsules encapsulating triethylene glycol dimethacrylate (TEGDMA) monomer and *N,N*-dihydroxyethyl-*p*-toluidine (DHEPT) tertiary amine accelerator as healing agents in self-healing dental composite [21–23]. Also, benzoyl peroxide (BPO) catalyst is an essential part of the resin matrix, functioning as a self-healing initiator, facilitating the chemical polymerization of the healing agents involved in the microcapsules [21–23]. A successful self-healing efficacy and recovery of the virgin fracture toughness (K_IC_) of approximately 65% have been reported [22]. The materials involved in self-healing dental composite have proven biocompatibility for dental use [22], however, further investigation is necessary before any in vivo studies in order to rule out the risk of cytotoxic unreacted free formaldehyde [46].

In the present study, the aims were to synthesise and characterise TEGDMA-DHEPT microcapsules, including optimisation of in situ emulsion polymerization of PUF shells, size analysis, optical and SEM imaging, and FT-IR of crushed microcapsules with BPO catalyst.

## Materials and methods

### Raw materials

The microcapsule shells were composed of urea, ammonium chloride, resorcinol and a 37% aqueous solution of formaldehyde (Sigma–Aldrich Company Ltd., Dorset, UK). The healing agent was a mixture of *N*,*N*-dihydroxyethyl-*p*-toluidine (DHEPT) amine (Esschem Europe Ltd., Seaham, UK) and triethylene glycol dimethacrylate (TEGDMA) monomer (Sigma–Aldrich Company Ltd., Dorset, UK). The surfactant used is poly (ethylene-alt-maleic anhydride) (EMA) (Average Mw 100,000–500.000 powder) (Sigma–Aldrich Company Ltd., Dorset, UK). pH regulators were hydrochloric acid (HCl) and sodium hydroxide (NaOH) 1 M solutions (Sigma–Aldrich Company Ltd., Dorset, UK). The chemical catalyst was benzoyl peroxide (BPO) (Sigma–Aldrich Company Ltd., Dorset, UK). All chemicals were analytical grade and used as received with no further purification.

### Synthesis of microcapsules

**Fig. 1:**
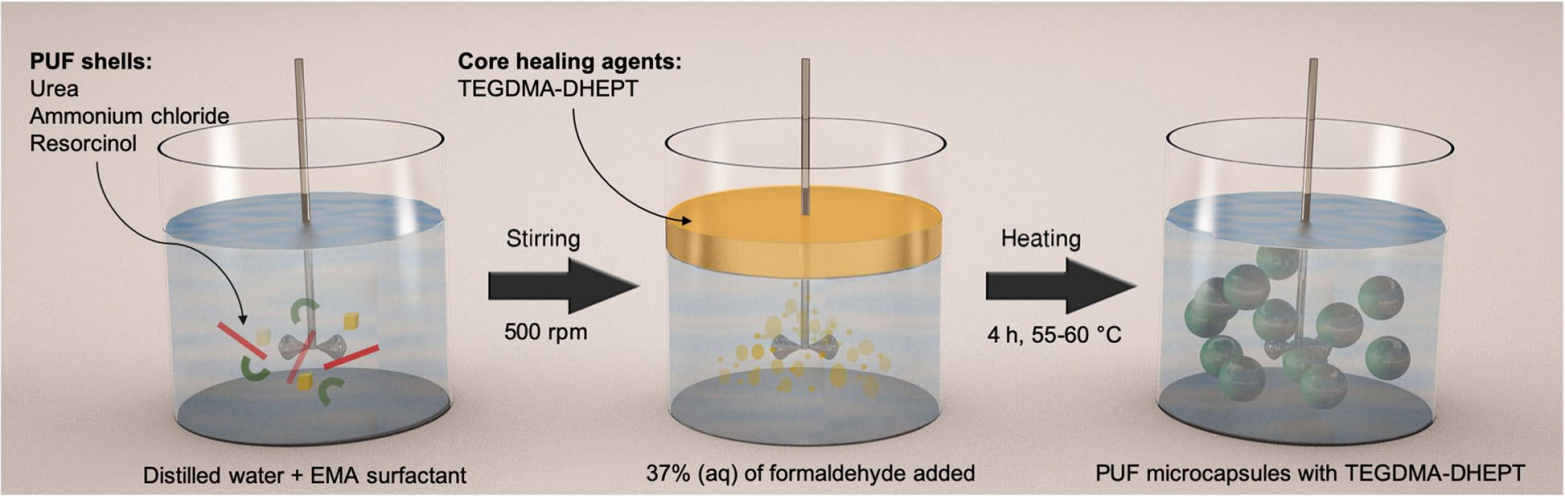
Encapsulation process of in situ emulsion polymerization of TEGDMA-DHEPT microcapsules

Microcapsules were prepared by in situ polymerization in an oil-in-water (O/W) emulsion (Fig. 1). At room temperature, 100 mL of distilled water and 26 mL of 2.5 wt% aqueous solution of poly (ethylene co-maleic anhydride) (EMA) copolymer were mixed into a 400 mL beaker flask. The beaker was held in a water bath on a hotplate with a digital display of temperature (Carousel tech stirring hotplate, Radleys, UK). A mechanical stirrer was used to agitate the solution, driving a four-bladed PTFE (40 mm) low-shear mixing propeller positioned just above the bottom of the beaker (Eurostar, IKA Ltd., UK). The stirring speed was set to 400 rpm. Then, 2.50 g urea, 0.25 g ammonium chloride and 0.25 g resorcinol were added into the flask. After dissolution of solids, the pH was checked and adjusted to 3.5 via drop-wise addition of 1 M NaOH solution.

Afterwards, the stirring speed was increased to 500 rpm. The healing liquid consisted of TEGDMA monomer and 1 wt% DHEPT amine. A slow stream of 60 mL of TEGDMA-DHEPT liquid was introduced to the reaction flask. After 10 min of stirring, a stabilised emulsion of fine TEGDMA-DHEPT droplets was formed. Then, 6.30 g of 37% aqueous solution of formaldehyde was added, and the flask was sealed with aluminium foil to prevent evaporation. The target temperature in the flask was 55–60 °C; an external temperature probe was placed in the bath for further confirmation.

The shell materials of the microcapsules were isothermally polymerised under continuous agitation. After 4 h, the suspension of microcapsules was left to cool to ambient temperature. Then, filtration through centrifugation and sedimentation were employed. The microcapsules suspension was centrifuged with distilled water. This process was repeated 5 times, for each 5 min cycle the solution was replaced with fresh distilled water in order to remove the remnant surfactant. Sieving of the microcapsules was undertaken with rinsing with distilled water repeatedly throughout the sieving process, microcapsules were then left to dry for 24 h.

### Microcapsules characterisation

#### Sizing of microcapsules

In order to separate the microcapsules according to size, a vibratory sieve shaker (Retsch® AS 200 digital, Retsch Limited, UK) was used. Four different sieves were used (45, 90, 150 and 300 μm pores sizes). The suspension of microcapsules was poured into the sieves and left for 30 min in the shaker (amplitude of 70% - 2.1 out of 3 mm). The microcapsules were allowed to dry overnight. Microcapsules were then collected with a plastic spatula into four glass bottles according to the sieve size and weighed.

#### Imaging analysis

An optical microscope (Leica DMI6000 B, Germany) was used to assess the encapsulation process, and to confirm the diameter of the microcapsules by image processing software (ImageJ, NIH Image). Following that, microcapsule surface morphology and further size analysis were conducted using a scanning electron microscope (Zeiss EVO60, Germany). A small number of microcapsules were spread onto adhesive tape and sputter-coated with gold (7 nm).

#### Degree of conversion of the microcapsules with the catalyst

An FT-IR spectrometer has been used to measure the degree of polymerization of TEGDMA-DHEPT healing agents with benzoyl peroxide catalyst (Avatar 360, Nicolete Analytical Instrument, Thermo Electron Corp., Cambridge, UK). A mixture of microcapsules and 0.5 wt% of BPO were broken in an agate mortar and pestle grinding bowl then placed and pressed in a PTFE disc mould (4 mm internal diameter, 0.5 mm height). Another PTFE disc mould (4 mm internal diameter, 2 mm height) was also used to test the polymerization of this mixture in 2 mm depth. The spectra were recorded over the range of 4000 to 400 cm^−1^ with 32 scans at a resolution of 4 cm^−1^, the degree of conversion (DC) of TEGDMA monomer was calculated from the peak intensity ratio of C=C at 1637 cm^−1^ against the internal standard peak of C=O at 1715 cm^−1^ immediately post-curing and 24 h after polymerization [47].

## Results

### Microcapsules characterisation

#### Size analysis

**Fig. 2:**
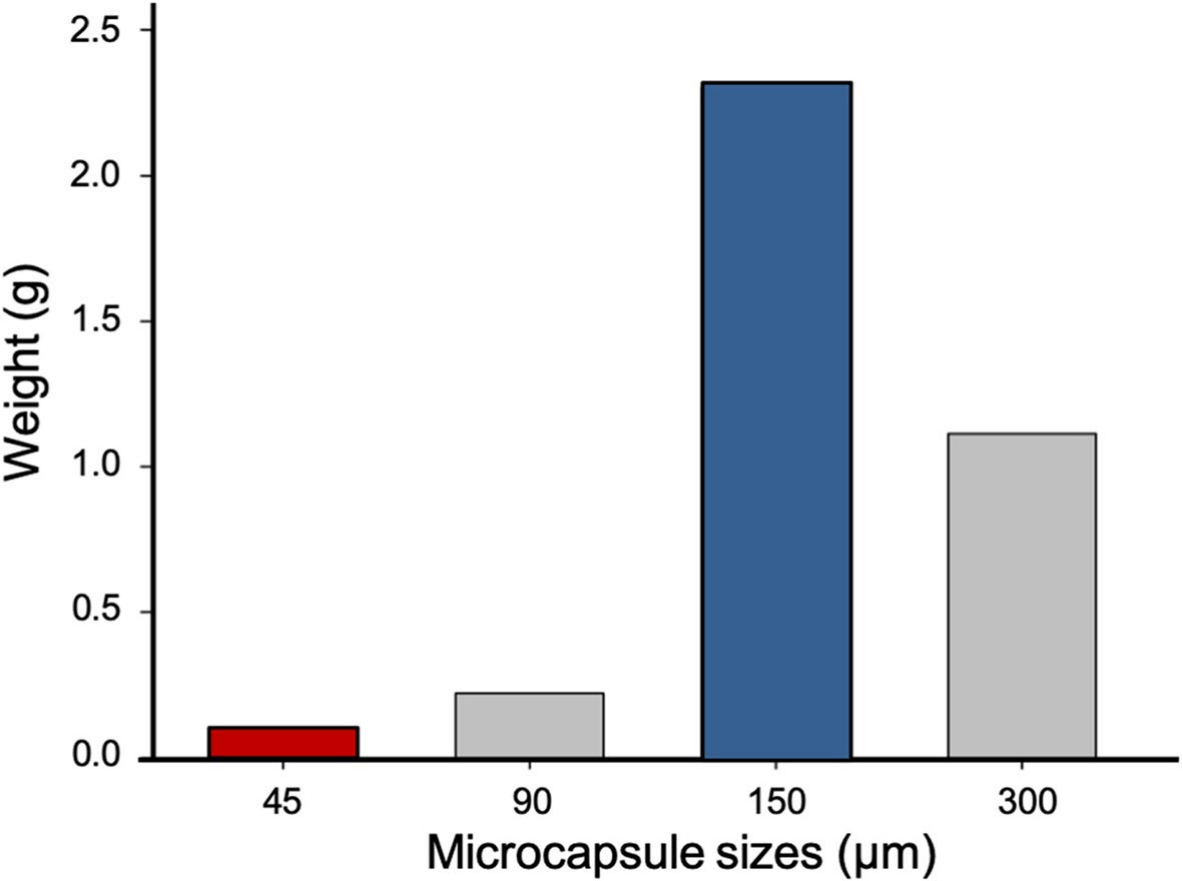
The weight distribution of different sizes of microcapsules

Synthesis of TEGDMA-DHEPT microcapsules was achieved by in situ polymerization to form poly(urea-formaldehyde) capsular shells. The distribution of microcapsule showed, a small number of microcapsules had a diameter between 45 μm to 90 μm. The size range of most microcapsules was 150 μm to 300 μm (Fig. 2).

#### Microcapsules imaging

**Fig. 3:**
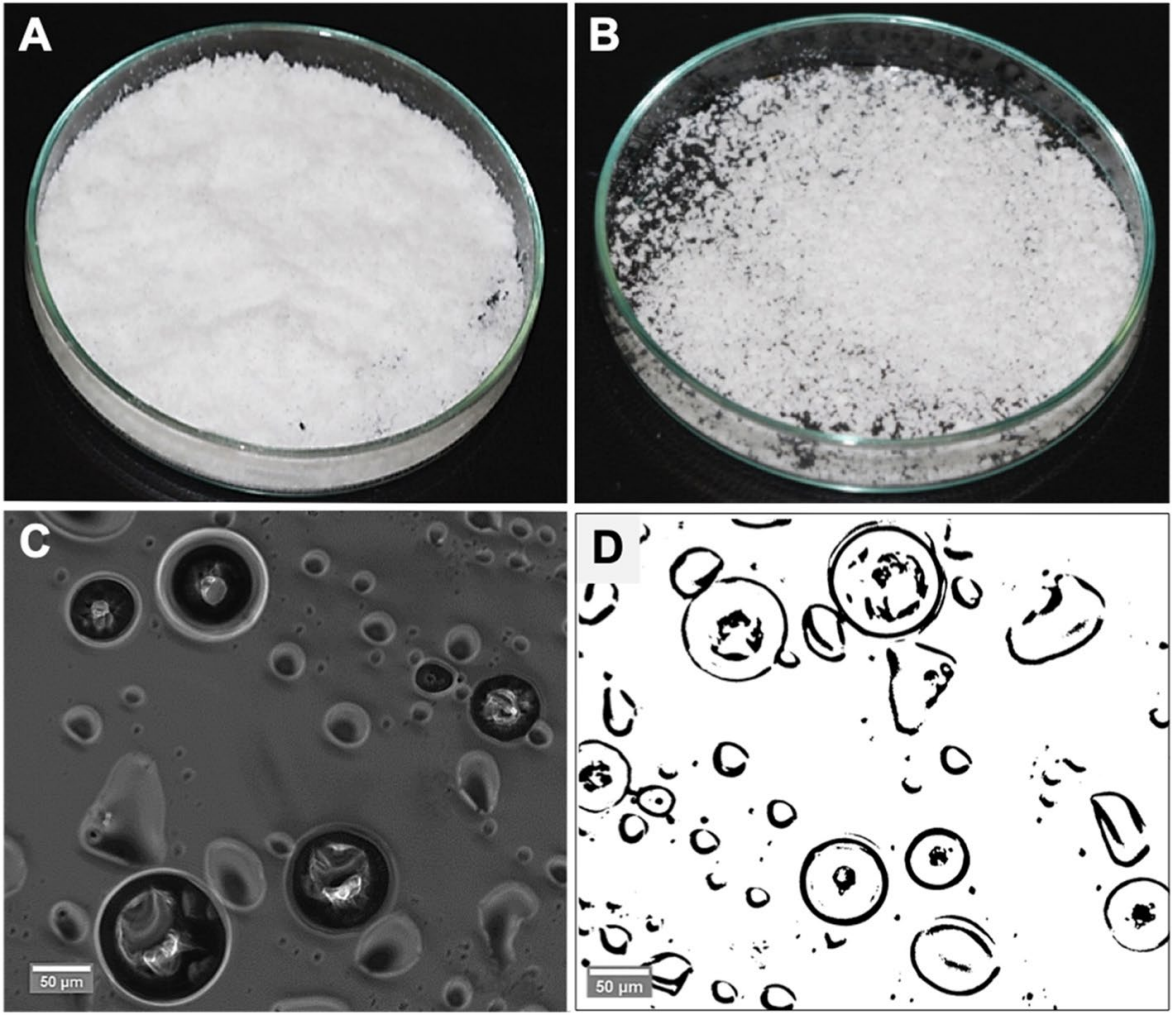
Self-healing TEGDMA-DHEPT microcapsules prepared via polymerization in situ;(**A**) Microcapsules presented as a free-flowing white powder, (**B**) poor quality microcapsules batch showing agglomerated and fused microparticles, (**C** and **D**) Optical microscope images presenting capsular shells as a dark outer ring, encapsulating the healing agents of a lighter shade opacity

After the filtration process, microcapsules presented as a white powder (Fig. 3, A). However, a number of the synthesised microcapsule batches showed as agglomerated and clustered particles (Fig. 3, B). Optical imaging further confirmed microcapsule sizes (as quantified in two dimensional planes of the sphere and averaged for each microcapsule); this revealed a diameter average of 120 ± 45 μm (*n*: 100). It is also showed an outer black ring that indicates shell formation and a moderately brighter area interiorly representing the encapsulated healing agents (Fig. 3, C and D).

**Fig. 4:**
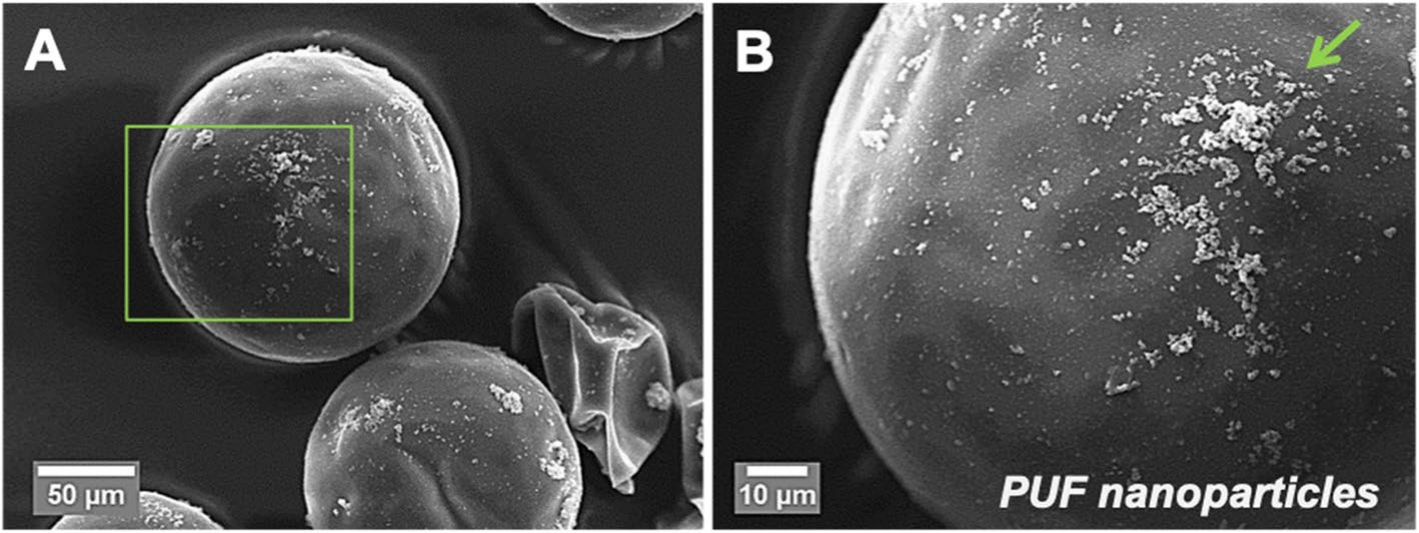
(**A**) SEM image of ≤150 μm microcapsules, also a ruptured microcapsule can be seen. (**B**) A higher magnification SEM image of the capsular shell, demonstrating a smooth outer shell with deposits of poly(urea-formaldehyde) nanoparticles

SEM showed a uniform external surface without voids detectable. The microcapsule diameter (Fig. 4, A), was estimated to be around 150 ± 50 μm (*n*: 100). The smooth external wall forms a rough surface morphology with the presence of PUF nanoparticles. (Fig. 4, B).

#### Degree of conversion of the microcapsules with the catalyst

The healing agents TEGDMA-DHEPT successfully polymerised in both 0.5 mm and 2 mm specimens after 24 h, showing a degree of conversion of 60.3 and 34.8% respectively. The analysis of the final polymer spectra confirmed the reactivity of the microcapsules after being crushed and mixed with BPO catalyst.

## Discussion

Self-healing composites and polymers, which incorporate microencapsulated healing liquids, have shown great promise in providing durable structural materials. These materials have the ability to repair crack damage and restore mechanical performance in polymeric materials, offering the potential for long-lasting functionality [3]. In the present work, TEGDMA-DHEPT microcapsules were successfully synthesised via emulsion polymerization to achieve polymeric capsular shells [36, 48]. Microcapsules were prepared by in situ polymerization; EMA acts as a surfactant, which helps to form an O/W emulsion, the oil being TEGDMA-DHEPT liquid. The microcapsules consisted of poly(urea-formaldehyde) shells with TEGDMA monomer and 1 wt% DHEPT amine as healing agents.

The final product of microcapsules was a free-flowing white powder, however, some agglomerated microcapsules were also found. Clustered or fused microcapsules are not ideal, but upon microcapsules dispersion into resin matrix, even distribution was achieved. One of the key factors for the creation of free-flowing microcapsules is the filtration process, which is a very sensitive and delicate procedure [36]. Poor filtration can result in an over-dryness of the microcapsules and may affect the permeability of the capsular shells by opening the shell pores. As a result, the microcapsules can become yellowish with time, due to healing liquid leaking through the shells resulting in the agglomeration and fusion of the microcapsules together.

Microcapsule size is an important factor to allow the encapsulation of sufficient healing agents to achieve self-healing capability in resin-based matrix. Smaller-sized microcapsules ≤70 μm will not be able to fill a crack due to the small amount of healing liquid. However, larger-sized microcapsules ≥300 μm will negatively impact the polymer matrix strength which may lead to voids following microcapsules rupture, resulting in deterioration of the mechanical properties of the polymeric material [49]. Microcapsule diameter can be controlled by stirring speed; an average diameter of 10–1000 μm was obtained by 200–2000 rpm [36]. A stirring speed of 500 rpm was set to obtain an average diameter of 150 μm microcapsules. Then, microcapsules were sieved using different sieves according to their capsular size, and showed the range was more of 150–300 μm microcapsule sizes. This technique was found to be a reliable and repeatable method for size sorting according to the anticipated application in resin-based materials. Optical or SEM microscopy can also be used to quantify microcapsule surface diameter although is time-consuming and requires the examination of several hundreds of particles to obtain statistically representative data. Optical and SEM imaging have also confirmed the microcapsules diameter ranging from 120 ± 45 μm to 150 ± 50 μm. Previous studies manufactured microcapsules with a similar diameter of 150–200 μm [36], or a smaller diameter of ≤70 μm microcapsules by using a higher stirring speed [22, 36].

Shell thickness of microcapsules is of a vital importance; it has been reported that if too thin, shells are more prone to breakdown during resin paste mixing, handling and packing [22]. However, if too thick, shells are more resistant to rupture upon composite fracture, which will prevent the delivery of healing liquid to the crack. Previous studies have reported an average shell thickness of 160–230 nm with DCPD or TEGDMA-DHEPT microcapsule, and showed that microcapsules ruptured upon composite fracture and self-healing capability was achieved [3, 22, 36].

SEM imaging showed that PUF nanoparticles existed on the external surface of the capsular shells. Deposition and aggregation of higher molecular weight pre-polymer on the outer PUF capsular surface during in situ polymerization resulted in a rough and porous surface morphology [36]. The inner shell surface was a smooth non-porous wall as a result of low molecular weight pre-polymer deposition. PUF nanoparticles on the outer surface of the microcapsules can facilitate mechanical interlocking interface (micromechanical retention) to the host resin polymer matrix upon photo activation (light-curing) [22]. This retention interface will allow the microcapsules to break when subjected to cracking. If the outer surface of the microcapsules had a smooth, non-porous morphology, the interlocking interface will be missing and the crack may bypass the microcapsules without breaking them, resulting in no healing liquid to fill the crack [22].

The ability of TEGDMA-DHEPT microcapsules to polymerise when crushed and mixed with 0.5 wt% benzoyl peroxide (BPO) catalyst was reported. BPO within the host polymeric material promotes the chemical reaction by free radical polymerization when it reacts with tertiary aromatic amine DHEPT in the healing liquid inside the ruptured microcapsules. An FT-IT spectral analysis was conducted within the range of 4000 to 400 cm^−1^, utilizing 32 scans at a resolution of 4 cm^−1^. To determine the degree of conversion (DC) of TEGDMA monomer, the peak intensity ratio of C=C at 1637 cm-1 was compared to the internal standard peak of C=O at 1715 cm-1. This comparison was done both immediately after curing and 24 hours after polymerization. In another study involving TEGDMA-DHEPT microcapsules, similar findings were observed [47]. A degree of conversion of 60.3% (in 0.5 mm depth) and 34.8% (in 2 mm depth) were obtained after 24 h self-cure at room temperature. Similar findings were reported by a recent study involving TEGDMA-DHEPT microcapsules crushed with 1 wt% BPO; a DC of 67.2% was reported [22]. In general, dimethacrylate resins have residual unsaturated monomers in the final polymerised resin matrix which can reach up to 43% [50]. In service, it is important to investigate how the reparative resin is released, rate of release, and the extent of mixing with BPO initiator. Also, the consequences of homopolymerization of TEGDMA as a reparative resin should be addressed, along with reaction kinetics of low molecular weight, high mobility resins and cyclisation reaction.

The current study used dental materials including TEGDMA monomer and DHEPT amine in the microcapsules and BPO catalyst within the resin composite matrix. The materials used have been approved by the Food and Drug Administration, and are available in commercial resin-based dental composites. Human gingival fibroblast cytotoxicity tests in vitro have shown that TEGDMA-DHEPT microcapsules exhibit an acceptable biocompatibility, hence the incorporation of microcapsules in resin does not drastically compromise cell viability [22], although the possibility of leakage of unreacted free formaldehyde from PUF shells should be taken under consideration for cytotoxicity testing.

## Conclusion

TEGDMA-DHEPT microcapsules were successfully synthesised by in situ polymerization of an O/W emulsion. The microcapsules have the ability to polymerise when they are ruptured and triggered by a BPO catalyst in the host composite. Microcapsule sizes ranged between 150 and 300 μm with an average of 120 ± 45 μm (*n*: 100). The morphology analysis showed a rough outer shell due to the presence of PUF nanoparticles. Microcapsules and BPO mixture showed a degree of conversion reached up to 60.3%, which confirms encapsulation of the healing agents and proves functionality of the microcapsules.

The incorporation of TEGDMA-DHEPT microcapsules in a self-healing dental composite model will be presented in our future studies. As yet, cytotoxicity testing should be conducted, considering the unreacted free formaldehyde in the PUF shells of microcapsules.

## Data Availability

All data generated or analysed during this study are included in this published article.
